# The role of the androgen receptor in ovarian cancer carcinogenesis and its clinical implications

**DOI:** 10.18632/oncotarget.12561

**Published:** 2016-10-11

**Authors:** Haiyan Zhu, Xuejie Zhu, Lihong Zheng, Xiaoli Hu, LuZhe Sun, Xueqiong Zhu

**Affiliations:** ^1^ Department of Obstetrics and Gynecology, the Second Affiliated Hospital of Wenzhou Medical University, Wenzhou, China; ^2^ Department of Gynecology, the First Affiliated Hospital of Wenzhou Medical University, Wenzhou, China; ^3^ Department of Cellular & Structural Biology, the University of Texas Health Science Center at San Antonio, San Antonio, TX, USA

**Keywords:** ovarian cancer, androgen receptor, clinical implications

## Abstract

Ovarian cancer is the major cause of death in women with gynecologic malignancies. There is emerging evidence that Androgen/androgen receptor (AR) signaling plays a critical role in the etiology and progression of this disease. Androgen receptor is frequently expressed in various subtypes of ovarian cancers and androgen/AR signaling has been shown to promote proliferation, migration, and invasion of ovarian cancer cells. Furthermore, shorter AR CAG repeats length and increased AR activity are associated with increased ovarian cancer risk and may be a useful prognosticator under certain circumstances. Here, we summarize current findings regarding the role of the AR in ovarian cancer and discuss agents that target this pathway as potential therapeutics for ovarian cancer.

## INTRODUCTION

Ovarian cancer is the most lethal gynecologic malignancy with an estimated 22,280 new cases and 14,240 deaths in 2016 in the United States [1]. Although many improvements have been made in surgical techniques and adjuvant therapies, the survival rate of ovarian cancer has changed little since platinum-based treatment was introduced over 30 years ago [2]. Poor prognosis of this malignancy is largely due to late detection, a lack of targeted therapies for advanced disease and manifesting chemoresistance [2, 3]. For these reasons, a better understanding of the molecular pathogenesis leading to ovarian cancer is of critical importance for the improvement of early-stage detection and new therapeutic targeting that may increase patient survival rate and well-being.

Since HA Risch (1998) first put forward a hypothesis for the pathogenesis of ovarian cancer relating to the role of androgens in stimulating epithelial cell proliferation [4], a large host of esoteric evidence has accumulated, including those of epidemiologic data, genetic elements and biologic nature, that points to an etiologic association between androgens and the development of ovarian cancer. Oral contraceptive use, tubal ligation, and hysterectomy are all characterized by decreased androgen levels, and reducing the risk of ovarian cancer, whereas polycystic ovarian syndrome, a hyper-androgenic condition, increases the risk of ovarian cancer [4]. An increased risk correlated with the use of exogenous androgenic agents provides further support for the androgen-epithelial ovarian cancer link [5]. For example, danazol use has demonstrated a 3.2-fold increased risk of ovarian cancers among women with endometriosis [6] and users of testosterone supplements are 3.7 times more likely to develop epithelial ovarian cancer when compared with the control group [7]. Additionally, data from animal models and cell lines implies that androgen treatment might stimulate the growth and/or advance the progression of ovarian cancer [8–10].

**Figure 1:**
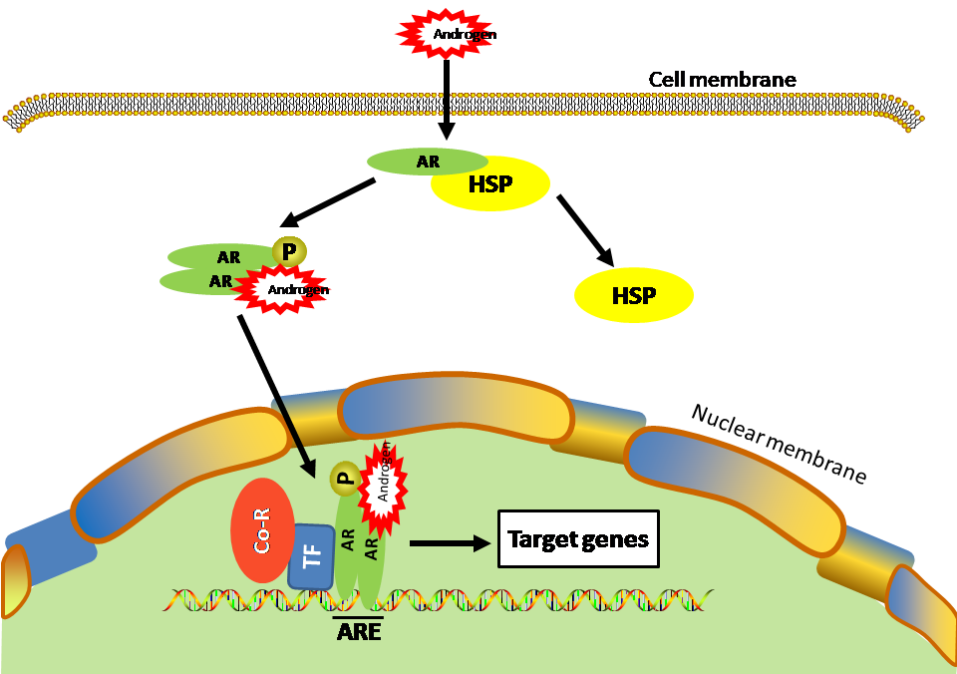
Ligand-dependent activation of AR Androgen binding to AR causes dissociation of the heat shock protein (HSP) from AR and subsequent translocation of AR into the nucleus where it binds to androgen-responsive element (ARE) in the chromosomes and regulates its target gene transcription with various transcription factors (TF) and co-regulators (Co-R).

The effects of androgens are mediated through the androgen receptor (AR), a steroid hormone receptor that belongs to the nuclear receptor superfamily [11]. In its basal state, AR is inactive and bound to heat shock proteins and other cellular chaperons. Activation by androgen hormone triggers a series of events, including dissociation from the heat shock proteins, phosphorylation, dimerization, and cumulating in nuclear translocation [12] (Figure 1). In the nucleus, the AR binds to specific DNA sequences known as androgen response elements in conjunction with various AR co-factors. The AR complex can therefore regulate the expression of genes that participate in various physiological and pathological functions [13, 14]. In addition to the canonical mechanism, AR can also be activated in the absence of androgens under certain pathological conditions [14, 15]. For example, AR can be activated in the absence of androgens by interleukin-6 (IL-6) in human prostate cancer cells [16]. AR is expressed in many cell types and the androgen/AR signaling has been found to promote tumorigenesis and metastasis in several types of cancers including prostate, bladder, kidney, lung, breast, liver and ovary [5, 17].

In this article, we provide a comprehensive overview on the role of AR in ovarian cancer carcinogenesis and progression. We also summarize and discuss key data describing clinical implications in ovarian cancer. A better understanding on the activity and clinical relevance of the steroid hormone receptor in ovarian carcinogenesis is essential for the development of potential prognostic marker and therapeutic target in this disease.

## AR EXPRESSION IN HUMAN OVARIAN CANCER

A large body of evidence has consistently vindicated over-expression of AR in ovarian cancers. In an early report, Hamilton et al. [18] demonstrated AR expression in ovarian cancers using ligand binding assays. Further studies showed AR expression was detected in about 90% of epithelial ovarian cancers by biochemical receptor assay [19] and 43.5-86% by immunohistochemistry. [20–23] Curiously in recent studies, AR expression has commonly been detected just as frequently as the estrogen receptor and more frequently than the progesterone receptor in ovarian cancer samples [21, 24].

About 90% of primary malignant ovarian tumors are epithelial ovarian cancer and can be further classified into five histopathological types: low-grade serous carcinomas, high-grade serous carcinoma, clear cell carcinoma, endometrioid carcinoma, and mucinous carcinoma [3, 5]. It is now recognized that risk factors, molecular events, prognostic markers, and therapeutic targets vary substantially across subtype in epithelial ovarian cancer. Some investigations sought to determine whether AR is divergently expressed in different histological subtypes of ovarian cancers. Cardillo et al. [23] reported that the incidence and expression levels of the AR varied widely in the different histological types of ovarian cancers. This observation is supported by Lee et al. [22] who reported that ARs were expressed in 43.7% of primary ovarian carcinoma samples but the most highly in serous (47.5%) carcinomas. Likewise, a study by de Toledo et al. [25] found that AR positivity expression tends to be more prevalent in serous than non-serous tumors. Sheach et al. [21] reported that AR scores showed no correlation with FIGO stage, residual disease or preoperative CA125 levels but a certain relationship with histological subtypes.

The association between AR expression and other clinical pathological characteristics such as tumor stages and grades has also been evaluated [23, 24, 26]. A study by Jonsson et al. [24] demonstrated that AR negativity was associated with high grade carcinomas. In contrast, four previous studies reported that no association was observed between AR scores and tumor FIGO stages in malignant ovarian tumors [21, 23, 25, 27]. Moreover, in a recent study, matched primary and metastatic serous ovarian cancer samples did not significantly differ with respect to nuclear AR levels [27]. Thus, AR is detected more so in serous than non-serous ovarian tumors but whether AR expression level is important for ovarian cancer progression remains to be determined.

## ROLE OF AR IN OVARIAN CARCINOGENESIS

### Promoting proliferation

A number of studies have shown that the ovarian surface epithelium is an androgen responsive tissue [9, 21, 28]. Edmondson et al. [8] first demonstrated that ovarian surface epithelium is an androgen responsive tissue and that androgens can cause an increase in proliferation and a decrease in cell death in eight primary cultures of human ovarian surface epithelium cells. Along the same line, Syed and colleagues found that testosterone and 5-dihydrotestos-terone significantly stimulated cell growth both in malignant and normal ovarian cell lines/cultures. This androgen-stimulated increase in growth was reversible by co-treatment of these cells with the anti-androgen 4-hydroxy flutamide [28]. In a recent study, androgen was reported to stimulate cell division and S-phase fraction in 11 primary culture ovarian cancer cell lines. Furthermore, the same study revealed a positive correlation between increased nuclear AR expression and increased S phase fraction changes in response to androgenic stimulation by immunohistochemistry [9]. There are also *in vivo* studies about androgen promoting tumor growth. Using guinea pigs, Silva et al. [29] found that testosterone treatment stimulated the growth of ovarian epithelial cells, resulting in benign cysts, small adenomas in the ovarian parenchyma, and papillomas on the ovarian surface. In a mouse model, Gruessner et al. [10] reported that androgen ablation of male mice led to a 24-fold decrease in tumor burden from serous ovarian cells. Moreover, the clinical evidence further confirmed this observation. Both danazol and testosterone have been reported to increase the risk of ovarian cancer. [6, 7].

Clearly, these findings reveal an important role for androgen/AR signaling in stimulating the growth and/or progression of ovarian cancers. One potential mechanism is by down-regulating their sensitivity to transforming growth factor-beta (TGF-beta), a potent inhibitor of epithelial cells, including malignant and nonmalignant ovarian cells [30–32]. We have recently demonstrated AR exerts its oncogenic effects in prostate tumors by down-regulating the type II receptor of TGF-beta, hence attenuating the tumor-suppressive activity of TGF-beta pathway in prostate cancer [33]. In ovarian cancer, androgen treatment down-regulated the expression of TGF-beta receptors and suppressed the growth inhibitory actions of TGF-beta [30–32]. Thus, we suspect that Androgen/AR signaling may promote ovarian cancer progression in part by decreasing TGF-beta receptor levels, thereby allowing ovarian cancer cells to escape TGF-beta growth inhibition.

Additionally, androgen-induced epithelial ovarian cancer proliferation may be partially due to the enhanced IL-6 and IL-8 expression, which could also promote epithelial ovarian cancer growth *via* activation of the AR gene promoter [34]. Thus, there may be a complex reciprocal regulation between AR signaling and IL-6/IL-8 during the carcinogenesis of ovarian cancer and further study is necessary to elucidate the underlying mechanisms. Recently, AR was reported to degrade cell cycle inhibitor p27 and down-regulate p21 expression in ovarian cancer [32] [35]. These studies argue that AR regulates cell cycle to control cellular proliferation. Additionally, Nourbakhsh et al. [36] showed that the androgens’ effect on ovarian cancer cells was associated with increased expression, activity, and phosphorylation of telomerase.

The epidermal growth factor receptor (EGFR) is over-expressed in 30-98% of epithelial ovarian carcinomas, and the activation of signaling cascades is linked to cell proliferation, migration and invasion, and angiogenesis, as well as resistance to cell apoptosis [37]. AR was reported to stimulate the synthesis of EGFR by autocrine or paracrine mechanism [38]. Crosstalk between EGFR and AR pathways has been shown to promote the progression of bladder cancer [39]. With respect to ovarian cancer, Ilekis et al. demonstrated an association between epidermal growth factor receptor and AR levels in ovarian cancer by western blot analysis of 60 serous cystadenocarcinomas [40]. At this time, it is unknown whether the cross-communication between EGFR and AR pathways functions in a similar manner to accelerate ovarian tumorigenesis

Using a cDNA microarray, Sheach et al. [21] identified 121 AR target genes with the majority being related to transcription, proliferation and G-protein signaling. Eight G-proteins were validated using quantitative reverse transcription-polymerase chain reaction, in which GTPase Rab35 was identified as the most differentially expressed gene upon androgen stimulation [21]. They also showed that Rab35 was expressed in the majority of ovarian tumors (95%) by immunohistochemical observation and its expression levels were correlated with AR levels. Thus, the authors speculated that Rab35 might be useful as a biomarker of AR function.

The AR also works in concert with AR coactivators to promote tumorgenesis. For instance, AR-associated protein 70 (ARA70) is a reported AR coactivator that enhances the transactivation potential of the AR up to 10-fold. ARA70 transcripts were negative in the normal ovarian surface epithelium, whereas it was highly expressed in 17 out of 20 ovarian carcinomas of various histological types [41]. P44/Mep50/WDR77 was identified as a subunit of the methylosome complex and lately characterized as a steroid receptor coactivator that enhanced AR as well as estrogen receptor-mediating transcriptional activity in a ligand-dependent manner. In his study, Ligr et al. [42] observed that p44 could serve as a coactivator of both AR and estrogen receptor in ovarian cells. Further, over-expression of nuclear-localized p44 stimulates proliferation and invasion in ovarian cancer cells in the presence of androgen or estrogen.

**Figure 2:**
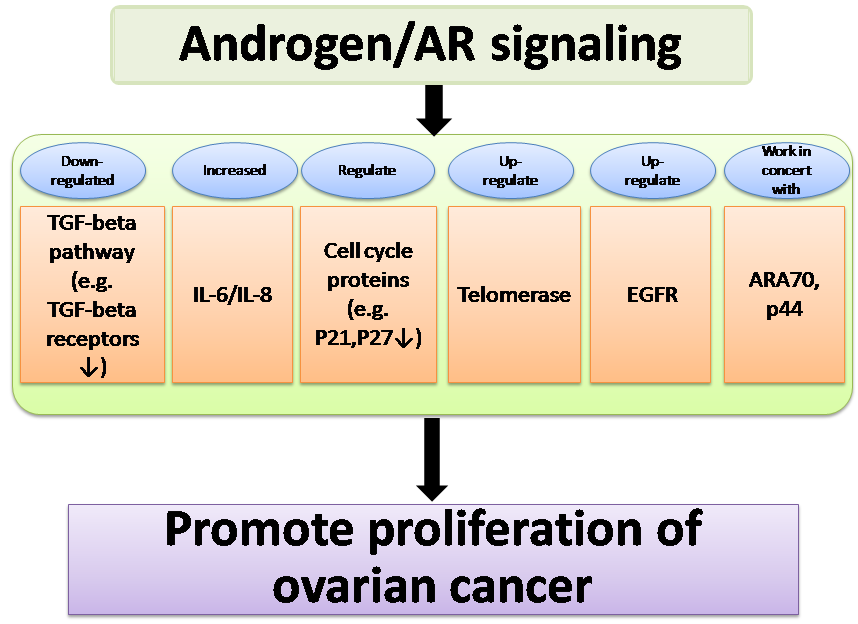
Summary of pathways affected by androgen/AR signaling, which acts in concert to promote proliferation of ovarian cancer Androgen/AR signaling promotes proliferation *via* interacting with a number of key components including the TGF-beta pathway, IL-6/IL-8, cell cycle regulators, telomerase, epidermal growth factor receptor, and AR coactivators.

In summary, these findings indicate that androgen/AR signaling promotes proliferation *via* interacting with a number of key components including the TGF-beta pathway, IL-6/IL-8, epidermal growth factor receptor, cell cycle regulators, and AR coactivators. (Figure 2).

### Stimulating cell migration and invasion

Although sparse, there is evidence from *in vitro* studies suggesting that androgens also contribute to the motility and invasion of epithelial ovarian cancer cell lines. Ligr et al. [42] tested the effects of androgen treatment on cell invasion in OVCAR-3 and SKOV-3 cell lines using an *in vitro* Matrigel invasion assay. The authors observed significantly increased invasiveness of the cells in media containing synthetic androgen when compared to cells in the hormone-free medium. Using two groups of mouse ovarian cancer cell lines displaying highly or moderately aggressive phenotypes *in vivo*, respectively, Du et al. [43] identified over-expressed AR and a 72 gene set representing potential biomarkers for moderately aggressive ovarian cancer. Accordingly, activation of AR may stimulate ovarian cancer cell migration and invasion resulting in a more aggressive phenotype.

We have recently established AR over-expressing OVCAR-3 and SKOV-3 ovarian cancer cell lines and evaluated their effect on proliferation and migration *in vitro*. We found that AR overexpression indeed promoted proliferation and migration of these ovarian cancer cell lines as determined by MTT proliferation and transwell migration assays. Our observations further support the idea that AR might function as a tumor promoter in ovarian cancer.

## CLINICAL SIGNIFICANCE OF AR IN OVARIAN CANCER

### Correlation of AR polymorphisms with ovarian cancer risk

The AR gene is located on the X chromosome, spans 90 kb and is composed of 8 exons [44]. Exon 1 contains two trinucleotide repeats that are polymorphic: a 9-39 CAG repeat (polyglutamine, polyQ) and a 14-27 GGN repeat (polyglycine, polyG). Taken together, these polymorphisms make up 90% of women with heterozygosity for the AR gene [44]. The most notable genetic factor influencing AR activity is the functional CAG repeats whose length is inversely proportional to its transactivation activity [45].. A number of studies have addressed the association between CAG repeat polymorphisms of the AR gene and ovarian cancer risk (Table 1). In an early study, Spurdle et al. [46] did not observe an association between ovarian cancer risk and AR exon 1 CAG(n) polymorphism, although they could not exclude threshold effects in a small subgroup. Dagan et al. [47] assessed the relevance of AR CAG repeat length and ovarian cancer risk in Jewish Israeli women who are BRCA1/2 mutation carriers, and null results were concluded, most likely due to the small sample size. However, two studies found that longer CAG repeats of the AR gene could increase the risk of ovarian cancer. Santarosa and colleagues [48] observed a 2.17-fold increased risk of ovarian cancer in Italy women with CAG(n) ≥ 22 and Terry et al. [45] reported a similar observation in a case-control study on population from either New Hampshire or eastern Massachusetts. On the contrary, several studies published recently found longer CAG repeat length could decrease the risk of ovarian cancer [49–53]. In a two-stage, case-control study from China, Meng et al. found that women with longer CAG_A repeats ( ≥ 22) had a 31% decreased risk of developing epithelial ovarian cancer compared to those with shorter CAG_A repeats ( < 22) [52]. Similar results were reported by Zhu et al., they found a 34% decreased epithelial ovarian cancer risk among women with longer AR CAG_A repeats ( ≥ 22) [53].

**Table 1:** Correlation of AR polymorphisms with ovarian cancer risk

Study	Population	Cases/Controls(n)	Findings
CAG repeat polymorphisms			
Spurdle et al. (2000)	Australia	319/ 853	No difference between case subjects and control subjects for the smaller, larger or average allele sizes of the CAG(n) genotype, before or after adjusting for age.
Dagan et al. (2002)	Jewish	41/78	No conclusive evidence of association between AR CAG repeat size and ovarian cancer risk in Jewish BRCA1/2 mutation carriers.
Santarosa et al. (2002)	Italy	121/100	An increase in the risk of ovarian cancer in women with CAGn >or=22 and a statistically significant trend towards an increased risk of ovarian cancer with increasing CAGn length.
Terry et al. (2005)	USA	987/1034	Carriage of two alleles with > or = 22 CAG repeats was associated with an increased risk of ovarian cancer compared with carriage of two alleles with <22 CAG repeats.
Schildkraut et al. (2007)	African American, Caucasian	594/681	No relationship observed between CAG repeat length and ovarian cancer among Caucasians, African Americans with a short repeat length on either allele was associated with a 2-fold increase in ovarian cancer risk.
Ludwig et al. (2009)	Poland	215/352	Longer AR (CAG)n repeat tracts decreased the risk of ovarian cancer.
Zhu et al. (2016)	China	1800/1800	Women with longer AR CAG repeats had a decreased EOC risk.
Meng et al. (2015)	China	1925/1900	Women with longer AR CAG repeats had a decreased risk of developing EOC.
GGN repeat polymorphisms			
Schildkraut et al. (2007)	African American, Caucasian	594/681	No relationship with the GGC repeat length polymorphisms was observed.
Meng et al. (2015)	China	1925/1900	No significant associations between GGN polymorphism and EOC risk.
Ludwig et al. (2009)	Poland	215/352	Longer AR (GGN)n repeat tracts decreased the risk of ovarian cancer.

AR, androgen receptor; EOC, epithelial ovarian cancer.

Overall, although the results of these epidemiologic studies were inconsistent, longer AR CAG repeats appeared to reduce the risk of ovarian cancer according to more recent, relatively large group data. Given that each additional AR CAG repeat resulted in a 2.5% decrease in AR transactivational activity [26] and androgen/AR-mediated growth stimulatory effects in ovarian cancer cell lines, these results further support the notion that a shorter AR CAG repeat, and increased AR activity may be involved in ovarian carcinogenesis.

The biological effects of the changing GGN repeat length have not been as widely studied as those of CAG. Studies found that the inverse association between AR protein levels and the GGN repeat length was related to the formation of a hairpin structure in AR mRNA, whose stability may interfere with translation [44]. The association between the AR GGN repeat polymorphism and ovarian cancer has been previously evaluated in three studies, however with conflicting results. One of the first hints of the possible relationship between GGN(n) repeat length polymorphism of the AR gene and ovarian cancer risk was observed by the group of Ludwig et al. [50]. The authors detected that increased GGN(n) repeat reduced the risk of ovarian cancer, but at the same time, another previous population-based study from North Carolina did not detect any relationship between the GGC repeat length polymorphisms of the AR gene and the risk of ovarian cancer [49]. Even more, a recent study among Chinese women didn't detect any significant associations between GGN polymorphism and epithelial ovarian cancer risk either [52]. Therefore, limitations in available data mean the importance of AR GGN repeat polymorphism in ovarian tumorigenesis requires further investigation.

## TARGETING AR IN OVARIAN CANCER

As alluded above, *in vitro* studies have suggested that androgen/AR signaling promotes tumor cell growth, invasion and survival in human ovarian cancer cell lines, which makes targeting AR a promising treatment strategy. Therapeutic targeting of AR has been employed since the 1990s; however, only a limited number of clinical trials have assessed the efficacy of anti-androgens in ovarian cancers so far. In an early report, Tumolo et al. [54] evaluated flutamide, a nonsteroidal drug with anti-androgen properties, in epithelial ovarian cancer patients pretreated with platinum-based chemotherapy. Of the 32 patients in this study, two patients responded to the treatment and nine patients had stable disease for a median of 24 weeks. The authors suggested that flutamide was an ineffective treatment for patients extensively pretreated with chemotherapy, and it was not devoid of side-effects (e.g. nineteen patients suffered side-effects including nausea and vomiting). In another phase II study of flutamide in ovarian cancers, Vassilomanolakis et al. [55] reported that only one partial response and two stable diseases were observed among the 23 patients evaluated, while the remaining 20 patients displayed progression of the disease within 3 months. In this trial, flutamide was well tolerated and showed a mild toxicity. More recently, bicalutamide, an oral nonsteroidal anti-androgen, was tested in a phase II study [56]. Thirty-five patients with epithelial ovarian cancer in a second or higher disease remission were treated with bicalutamide and goserelin, a GNRH agonist, but this did not appear to prolong progression-free survival in this group of patients. Although clinical responses to anti-androgen are variable, there are clearly subgroups of patients that respond very well to this kind of treatment. Thus, it is critical to be able to identify subgroups of patients responsive to anti-androgen therapy. There are a few studies that have attempted to identify biomarkers capable of predicting an anti-androgen treatment response in ovarian cancer. Elattar et al. [9] reported that nuclear expression of AR might be a viable biomarker for androgen sensitivity and Gruessner et al. found that patients with high CSF-1 and ErbB4 expression in the ovarian stroma were highly sensitive to flutamide [10]. However, these studies are still in their infancy and should be expanded upon in the future.

Taken together, this data suggests that anti-androgen in ovarian cancer may have certain effects under viable application conditions [12]. However, clinical trials testing anti-androgen therapy in ovarian cancer to date are relatively small and nonrandomized. Moreover, the agents that have been tested for AR blockade efficacy in epithelial ovarian cancer, including flutamide and bicalutamide are known to be weak AR antagonists and most clinical trials assessing anti-AR strategies in epithelial ovarian cancer have not measured AR activity [5, 12]; therefore, the results cannot be properly appraised for a solid conclusion. Further studies of anti-androgen treatment in patients with ovarian cancer using larger sample sizes, randomized design, validated biomarkers, and novel anti-androgens, and are needed. For example, abiraterone (an androgen biosynthesis inhibitor) and enzalutamide (an AR blocker) are both approved for treatment of castration resistant prostate cancer and have demonstrated a survival advantage in chemotherapy-naive and chemotherapy refractory prostate cancer patients [57]. Furthermore, AR-V7 has been used as a potential predictive biomarker to identify prostate cancer patients that will not benefit from these targeted agents [58]. More recently, Δ4-abiraterone, which is structurally similar to endogenous steroidal 5α-reductase substrates, was shown to be more clinically effective than abirateone treatment and provided an additional explanation for abiraterone's clinical activity [59]. Results with such novel anti-androgens in prostate cancer encourage further exploration of the benefit of anti-androgen therapy in ovarian cancer [12].

## PREDICTIVE VALUE OF AR FOR CHEMOSENSITIVITY IN OVARIAN CANCER

Several studies suggest that AR may be a valuable biomarker in predicting and evaluating patient's responses to chemotherapy treatments [60]. Elattar et al. [9] noticed that nuclear and cytoplasmic AR expression was decreased significantly in post-chemotherapy histological samples from 29 epithelial ovarian cancer patients when compared with the pre-chemotherapy counterparts. Using a microarray analysis, Sun et al. [61] identified 112 highly up-regulated genes in taxol-resistant SKOV3 ovarian cancer cells, in which 30 genes formed an interaction network involving AR bioactivity. Silencing AR using RNA interference produced a 3-fold sensitization to taxol in taxol-resistant cells, a response similar to which produced by silencing ABCB1 (multidrug resistance protein 1). Silencing AR also down-regulated the expression of prominent taxol-resistant gene candidates including ABCB1, ABCB6, ABCG2, BMP5, FAT3, FGFR2, H1F0, SRCRB4D, and TMPRSS15. In contrast, AR activation using the agonist 5-dihydrotestos-terone un-regulated the expression of these target genes. Individually silencing seven out of nine (78%) AR-regulated taxol-resistant genes sensitized taxol-resistant cells to taxol. Inhibition of AKT and JNK cellular kinases using chemical inhibitors caused a dramatic suppression of AR expression. It is clear that AR represents a critical driver of gene expression involved in taxol-resistance [62]. Therefore, it is plausible that AR expression is involved in response to chemotherapy and could be a potential biomarker to predict the patient outcome to treatment.

## PROGNOSTIC SIGNIFICANCE OF AR IN OVARIAN CANCER

Given the tumor-promoting role of AR, several other studies evaluated the potential prognostic value of AR in ovarian cancer patients (Table 2). In a prospective multicenter randomized controlled phase II trial, van Kruchten et al. [63] observed a trend towards AR-positivity associated with decreased overall survival in 121 epithelial ovarian cancer patients, but not reaching statistical significance (*P* = 0.10). Nevertheless, Lee et al. and Toledo et al. pointed out that AR expression was not associated with survival [22, 25]. Conversely, Nodin et al. [64] reported that AR positivity predicted a prolonged disease-specific survival in the serous subtype of epithelial ovarian cancer. This data is supported by two recent studies: one using the TCGA RNA-sequencing data, demonstrated that low AR expression was associated with shorter overall survival [65] in high-grade serous ovarian carcinoma while the other using immunohistochemical staining in 118 serous and endometrioid ovarian cancers reported that high level of AR was correlated with improved 5-year progression-free and overall survival [24]. Collectively, these results support the notion that increased AR expression tends to predict a favorable prognosis in epithelial ovarian cancer, especially in serous carcinoma of the ovary.

**Table 2:** Prognostic significance of AR and AR alleles in ovarian cancer

Study	No. of patients	Disease type	Findings
Lee et al.(2005)	322	Primary ovarian carcinoma	AR expression was not associated with survival.
de Toledo et al. (2014)	152	Primary EOC	AR expression was associated with neither pathological features of EOC nor disease-free/overall survival.
van Kruchten et al.(2015)	121	EOC	AR-positivity was associated with decreased overall survival, but not reaching a statistical significance.
Nodin et al.(2010)	154	EOC	AR expression was not related to prognosis in the whole cohort, whereas was associated with a prolonged disease specific survival in the serous subtype.
Martins et al. (2014)	255	HGSOC	Low AR expression was associated with shorter overall survival.
Jonsson et al. (2015)	118	Serous and endometrioid ovarian cancers	Expression of AR protein was associated with improved 5-year progression-free and overall survival.
Li et al. (2010)	62	EOC	Patients with a short AR CAG allele length did not demonstrate statistical differences in progression-free survival or overall survival.
Li et al. (2003)	77	EOC	EOC patients with less CAG repeats (≤19) experienced shorter time to recurrence and a decreased overall survival.
Ludwig et al. (2009)	69	EOC	In all 69 patients, longer AR (CAG)n repeats decreased the risk of recurrence by 55%. In the group with TP53 accumulation, longerAR (CAG)n repeats decreased the risk of recurrence and death.

AR, androgen receptor; EOC, epithelial ovarian cancer; HGSOC, high-grade serous ovarian carcinoma.

In addition to AR expression, the effects of AR alleles on prognosis have also been evaluated. Li et al. [66] reported that epithelial ovarian cancer patients with less CAG repeats (≤ 19) experienced shorter time to recurrence and had a decreased overall survival, suggesting that short CAG(n) repeat in AR allele implied a significantly worse overall survival in epithelial ovarian carcinoma. Similar results were reported by Ludwig et al. [50], longer AR (CAG)n repeats decreased the risk of recurrence by 55% with a much stronger association in the TP53 accumulation patients. This data suggests that short AR allele lengths may be a potential marker for poor prognostic factor in epithelial ovarian carcinoma, especially in patients with TP53 dysfunctional ovarian cancers. One possible reason may be shorter CAG repeat alleles promote aggressive ovarian cancer phenotype through variation of epidermal growth factor receptor signaling [26]. However, when evaluating the AR CAG allele length of clinical outcome in BRCA1/2 mutation positive women with ovarian cancer, Li et al. failed to find any association of AR CAG allele length with clinical outcome [67]. In summary, while AR alleles could be a useful ovarian cancer prognosticator under certain circumstances, the ovarian cancer subtypes and/or pathological conditions where it may best apply need to be further elucidated.

## CONCLUSIONS

AR is expressed in a majority of human ovarian cancers and androgen/AR signaling stimulates proliferation, cell migration and invasion of ovarian cancer cells. Shorter AR CAG repeats length and increased AR activity predict a high risk of ovarian cancer. Clinical data suggests that anti-androgen treatment in ovarian cancer may be effective for some patients. Since agents used for AR blockade in ovarian cancers are known to be weak AR antagonists, novel promising treatments that are more potent suppressors of the AR axis should be evaluated in clinical trials for ovarian cancers. Further, the use of tumor genomic expression profiling and the detection of patient AR response biomarkers may be very important in selecting appropriate patients for anti-androgen therapeutics, monitoring the bioactivity of new drug candidates, and predicting therapy outcomes and adverse events. In addition, AR expression is involved in response to chemotherapy and could be a potential biomarker to predict patient outcome to treatment. Increased AR expression likely predicts a favorable prognosis in epithelial ovarian cancer.
